# Does the type of anesthesia really affect the recurrence-free survival after breast cancer surgery?

**DOI:** 10.18632/oncotarget.21014

**Published:** 2017-09-18

**Authors:** Myoung Hwa Kim, Dong Wook Kim, Joo Heung Kim, Ki Young Lee, Seho Park, Young Chul Yoo

**Affiliations:** ^1^ Department of Anesthesiology and Pain Medicine, Yonsei University College of Medicine, Seodaemun-gu, Seoul, Republic of Korea; ^2^ Anesthesia and Pain Research Institute, Yonsei University College of Medicine, Seodaemun-gu, Seoul, Republic of Korea; ^3^ Department of Policy Research Affairs, National Health Insurance Service Ilsan Hospital, Gyeonggi-do, Goyang, Republic of Korea; ^4^ Division of Breast Surgery, Department of Surgery, Yonsei University College of Medicine, Seodaemun-gu, Seoul, Republic of Korea

**Keywords:** anesthesia, breast cancer, propofol, recurrence, volatile agent

## Abstract

**Background:**

Although previous studies have suggested that propofol inhibits cancer recurrence and metastasis, the association between anesthetic agents and the recurrence of breast cancer has not been clearly investigated. We compared total intravenous anesthesia and balanced anesthesia with volatile agents to investigate the differences in their effects on recurrence-free survival and overall survival after breast cancer surgery.

**Materials and Methods:**

The electronic medical records of 2,729 patients who underwent breast cancer surgery between November 2005 and December 2010 were retrospectively reviewed to analyze the factors associated with recurrence-free survival after surgery. Cox proportional hazards models were used to identify the risk factors for cancer recurrence and overall mortality after breast cancer surgery.

**Results:**

Data from 2,645 patients were finally analyzed. The recurrence-free survival rate in this study was 91.2%. Tumor-node-metastasis staging exhibited the strongest association with breast cancer recurrence. However, we were unable to identify significant differences between the preventive effects of total intravenous anesthesia and those of volatile agents on postoperative breast cancer recurrence using Cox regression analyses and propensity score matching. Furthermore, the survival probability with regard to postoperative recurrence and mortality showed no significant differences among anesthetic agents.

**Conclusions:**

Our findings suggest that the effects of total intravenous anesthesia are comparable with those of volatile agents with regard to postoperative recurrence-free survival and overall survival in patients with breast cancer.

## INTRODUCTION

Breast cancer is the most common malignancy and the leading cause of cancer-related death in women. Several factors may be related to breast cancer recurrence or metastasis, including surgery, stress hormones, immune-suppression, acute postoperative pain, and opioid analgesics [1–3]. After reports showing that cancer outcomes could be altered by the choice of anesthetic agent during surgery [4], this field has been emerging as an area of interest. An animal study demonstrated that opioids, volatile anesthetics, thiopental, and ketamine, but not propofol, could inhibit natural killer (NK) cell activity and cause cancer metastasis [4]. Preliminary data from an ongoing prospective randomized controlled trial for radical cystectomy support these findings; improved immune function was found in patients who received total intravenous anesthesia (TIVA) during surgery. In particular, the patients demonstrated enhanced cell-mediated immunity [5]. Recently, Forget et al. suggested that perioperative ketorolac significantly reduces the relapse of breast cancer [6], and that inflammation might be the precipitating factor and common denominator in early relapses [7].

However, several studies have recently questioned the influence of the anesthetic agent and technique on long-term tumor growth [8, 9]. Moreover, a systematic review conducted in 2014 concluded that there is insufficient data to make any firm conclusions [10].

Therefore, we conducted the present study to compare TIVA and balanced anesthesia with volatile agents with regard to their effects on the postoperative recurrence-free survival and overall survival of patients with breast cancer.

## RESULTS

### Study population, demographic data and perioperative characteristics

From November 2005 to December 2010, 2,729 patients underwent surgery following breast cancer diagnosis. Among these patients, 63 cases of multiple surgery and 21 cases with unclear anesthetic methods were excluded. The remaining 2,645 cases, which included 2,589 in the balanced anesthesia group (Sevoflurane group: 1613, Desflurane group: 664, Isoflurane group: 271, Enflurane group: 41) and 56 in the TIVA group, were analyzed for this study. The mean (standard deviation) follow-up duration of our papulation was 70.1 (23.2) months. The mean recurrence-free survival duration was 67.6 and 74.4 months with volatile agents and TIVA, respectively. The corresponding overall survival duration was 69.9 and 77.1 months with volatile agents and TIVA, respectively. Table 1 presents a comparison of characteristics between patients with and patients without breast cancer recurrence. There were no significant differences in baseline demographic data and anesthetic data between the two groups. With regard to the surgical factors, the surgery type, tumor-node-metastasis (TNM) stage, estrogen and progesterone receptor (ER and PR) status, histological type, and chemotherapy use showed significant differences between groups.

**Table 1:** Comparison of characteristics between patients with breast cancer recurrence and those without breast cancer recurrence

	No recurrence group (*N* = 2412)	Recurrence group (*N* = 233)	*P*-value
**Demographic data**			
**Age (years)**	50.1 (10.2)	48.9 (10.5)	0.072
**BMI (kg/m^2^)**	23.3 (3.1)	23.3 (2.9)	0.962
**Comorbidity**			
HTN	478 (19.8)	44 (18.9)	0.796
DM	170 (7.1)	18 (7.7)	0.689
Cardiac disease	61 (2.5)	6 (2.6)	> 0.999
Pulmonary disease	53 (2.2)	6 (2.6)	0.643
Endocrine disease	115 (4.8)	11 (4.7)	> 0.999
Renal disease	18 (0.7)	1 (0.4)	> 0.999
Liver disease	16 (0.7)	3 (1.3)	0.231
Neurological disease	41 (1.7)	4 (1.7)	> 0.999
Others	22 (0.9)	2 (0.9)	> 0.999
**Anesthetic factors**			
**Type of anesthetic agent**			0.301
Sevoflurane	1480 (61.4)	133 (57.1)	
Desflurane	606 (25.1)	58 (24.9)	
Isoflurane	241 (10.0)	30 (12.9)	
Enflurane	35 (1.5)	6 (2.6)	
TIVA	50 (2.1)	6 (2.6)	
**Induction agents**			0.253
Propofol	1850 (76.7)	187 (80.3)	
Barbiturate	562 (23.3)	42 (19.7)	
**N_2_O**	178 (7.4)	26 (11.2)	0.052
**Muscle relaxants**			
Atracurium	95 (3.9)	12 (5.2)	0.382
Vecuronium	1 (0)	0 (0)	> 0.999
Rocuronium	2318 (96.1)	221 (94.8)	0.379
Premedication*	1612 (66.8)	151 (64.8)	0.561
**Antiemetic**	2067 (85.7)	196 (84.1)	0.495
**Rescue analgesics**	2309 (95.7)	229 (98.3)	0.056
**Hypertensive events**	145 (6.0)	17 (7.3)	0.394
**Hypotensive events**	336 (13.9)	35 (15.0)	0.622
**Colloid administration**	39 (1.6)	4 (3.8)	0.788
**RBC transfusion**	10 (0.4)	2 (0.9)	0.286
**Surgical factors**			
**Surgery**			< 0.001
Breast-conserving	1194 (49.5)	63 (27.0)	
Mastectomy	1218 (50.5)	170 (73.0)	
**Surgical duration (min)**	207.2 (131.0)	212.5 (108.5)	0.487
**TNM stage**			< 0.001
1	1201 (49.8)	46 (19.7)	
2	886 (36.7)	85 (36.5)	
3	325 (13.5)	102 (43.8)	
**Receptors**			
Estrogen	1699 (70.4)	137 (58.8)	< 0.001
Progesterone	1541 (63.9)	123 (52.8)	0.001
HER2	658 (27.3)	62 (26.6)	0.878
**Histological analysis**			< 0.001
Well differentiated	539 (22.3)	20 (8.6)	
Moderately differentiated	1063 (44.1)	110 (47.2)	
Poorly differentiated	54 (22.6)	91 (39.1)	
Others	266 (11.0)	12 (5.2)	
**Tumor types**			0.311
IDC	2114 (87.6)	212 (91.0)	
ILC	90 (3.7)	5 (2.1)	
Others	208 (8.6)	16 (6.9)	
**Chemotherapy**	1605 (66.5)	204 (87.6)	< 0.001
**Radiotherapy**	1546 (64.1)	160 (68.7)	0.174

Data are presented as mean (standard deviation), or number (proportion, %).

BMI: body mass index, HTN: hypertension, DM: diabetes mellitus, TIVA: total intravenous anesthesia (with propofol), N_2_O: nitrous oxide, RBC: red blood cell, TNM: tumor–node–metastasis, HER2: human epidermal growth factor receptor 2, IDC: invasive ductal carcinoma, ILC: invasive lobular carcinoma

Premedication*: Midazolam 0.03 mg kg^-1^ was administered.

### Association between anesthetic agents and cancer recurrence after surgery for breast cancer

Table 2 shows the findings of the Cox regression analyses for factors increasing postoperative breast cancer recurrence. According to the multivariate Cox regression analyses, there was no difference in the recurrence-free survival between TIVA and balanced anesthesia. Recurrence of cancer was significantly related to surgical procedure, higher cancer stage, ER-positive cancer cells, and implementation of chemotherapy.

**Table 2:** Findings of univariate and multivariate Cox regression analyses for factors associated with the cancer recurrence after surgery for breast cancer

Parameters		Univariate				Multivariate			
		HR	95% CI		*P*-value	HR	95% CI		*P*-value
**Age (yr)**	< 40	1(ref)				1 (ref)			
	40–50	0.682	0.475	0.980	0.038	0.79	0.548	1.138	0.206
	50–60	0.724	0.495	1.057	0.094	0.771	0.527	1.129	0.182
	60–70	0.674	0.426	1.064	0.091	0.763	0.481	1.21	0.250
	> 70	0.572	0.258	1.269	0.170	0.7	0.306	1.603	0.399
**BMI**	< 18.5	1.039	0.526	2.053	0.912	0.845	0.427	1.674	0.630
	18.5–23	1 (ref)				1 (ref)			
	23–25	1.027	0.740	1.425	0.875	1.104	0.794	1.534	0.557
	25–30	1.053	0.764	1.450	0.753	1.008	0.731	1.39	0.961
	> 30	0.850	0.374	1.936	0.699	0.774	0.338	1.774	0.545
**Type of Anesthetic agents**	TIVA	1 (ref)				1 (ref)			
	Volatile agents	0.857	0.380	1.929	0.709	1.136	0.496	2.597	0.763
**Antiemetic**	No	1(ref)				1 (ref)			
	Yes	1.013	0.711	1.444	0.942	0.991	0.69	1.422	0.961
**Rescue Analgesics**	No	1(ref)				1(ref)			
	Yes	1.805	0.578	5.639	0.310	1.361	0.429	4.311	0.601
**Surgical procedure**	BCS	1(ref)				1 (ref)			
	Mastectomy	2.533	1.897	3.383	< 0.001	1.888	1.397	2.552	< 0.001
**TNM stage**	1	1(ref)				1 (ref)			
	2	2.404	1.679	3.441	< 0.001	1.806	1.182	2.76	0.006
	3	7.477	5.278	10.591	< 0.001	5.149	3.332	7.957	< 0.001
**ER**	Negative	1(ref)				1 (ref)			
	Positive	0.569	0.438	0.738	< 0.001	0.628	0.479	0.822	0.007
**Chemotherapy**	No	1 (ref)				1 (ref)			
	Yes	3.289	2.229	4.853	< 0.001	1.303	0.792	2.142	0.298

CI: confidence interval, OR: odds ratio, BMI: body mass index, TIVA: total intravenous anesthesia (with propofol), BCS: breast conserving surgery, TNM: tumor–node–metastasis, ER: estrogen receptor

**Figure 1:**
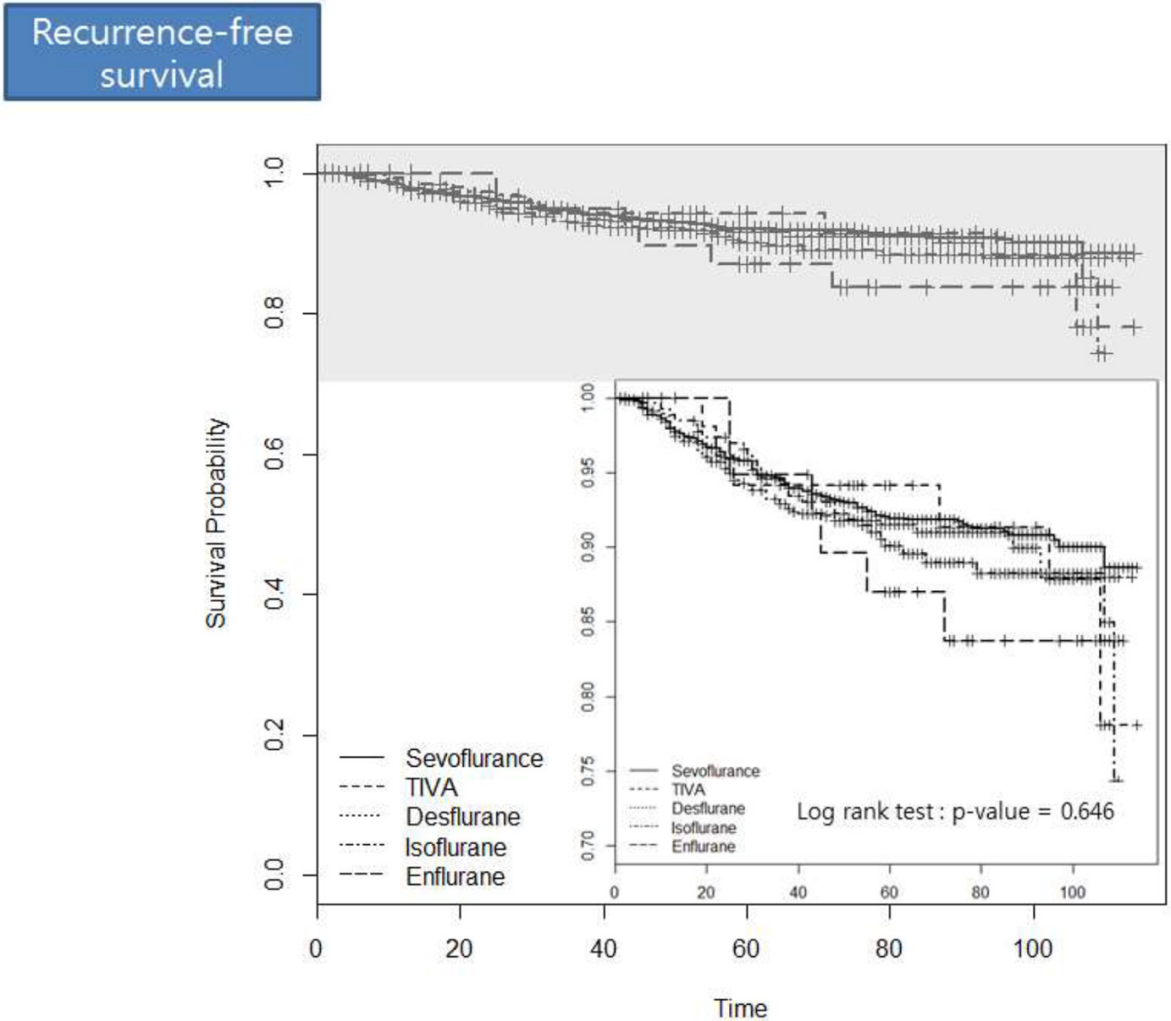
The survival probability for each anesthetic agent with regard to the recurrence after breast cancer surgery

Table 3 describes the sensitivity analysis for our primary findings. There were no differences in the recurrence after breast cancer surgery between TIVA and the volatile anesthetics. Next, in our 1-to-5 propensity score-matched analysis, there was also no association of TIVA or any of the volatile agents with postoperative recurrence. Figure 1 demonstrates the survival probability for each anesthetic agent with regard to recurrence-free survival after breast cancer surgery. Log-rank tests showed no significant differences between agents (*P* = 0.646).

**Table 3:** Association between anesthetic agents and postoperative cancer recurrence in patients with breast cancer after multivariate Cox regression analyses and propensity score matching

Parameters		HR	95% CI		*P*-value
**Type of Anesthetic agent**	TIVA	1 (ref)			
	Sevoflurane	1.066	0.463	2.452	0.881
	Desflurane	1.231	0.520	2.912	0.637
	Isoflurane	1.330	0.544	3.256	0.532
	Enflurane	1.451	0.459	4.594	0.526
**1:5 matching**	TIVA	1 (ref)			
	Sevoflurane	1.152	0.382	3.473	0.802
**1:5 matching**	TIVA	1 (ref)			
	Desflurane	1.233	0.314	4.839	0.764
**1:5 matching**	TIVA	1 (ref)			
	Isoflurane	1.829	0.306	10.946	0.508
**1:5 matching**	TIVA	1 (ref)			
	Enflurane	-	-	-	-

HR: hazard ratio, CI: confidence interval, TIVA: total intravenous anesthesia (with propofol)

*Multivariate Cox regression analyses were adjusted by age, body mass index (BMI), antiemetic, rescue analgesics, surgery type, tumor–node–metastasis (TNM) stage, estrogen receptor status, and chemotherapy use. The statistical significance of differences in the risk of recurrence between anesthetics was tested prior to matching, and the results demonstrated that, when propofol was used as a reference, the other anesthetics showed increased recurrence risk. However, the differences were not statistically significant.

**For more sensitive and precise analysis, the relative risk of postoperative recurrence after breast cancer surgery was calculated after 1:5 propensity score matching after adjustment for age, BMI, hypertension, diabetic mellitus, cardiac disease, pulmonary disease, endocrinal disease, renal disease, liver disease, neurological disease, and TNM stage. The results showed no statistically significant differences between TIVA and volatile anesthetics. Because the frequency of enflurane use was low, event occurrence after matching did not meet the minimum number requirement. Therefore, its relative risk was not calculated.

### Association between anesthetic agents and overall mortality after surgery for breast cancer

**Figure 2:**
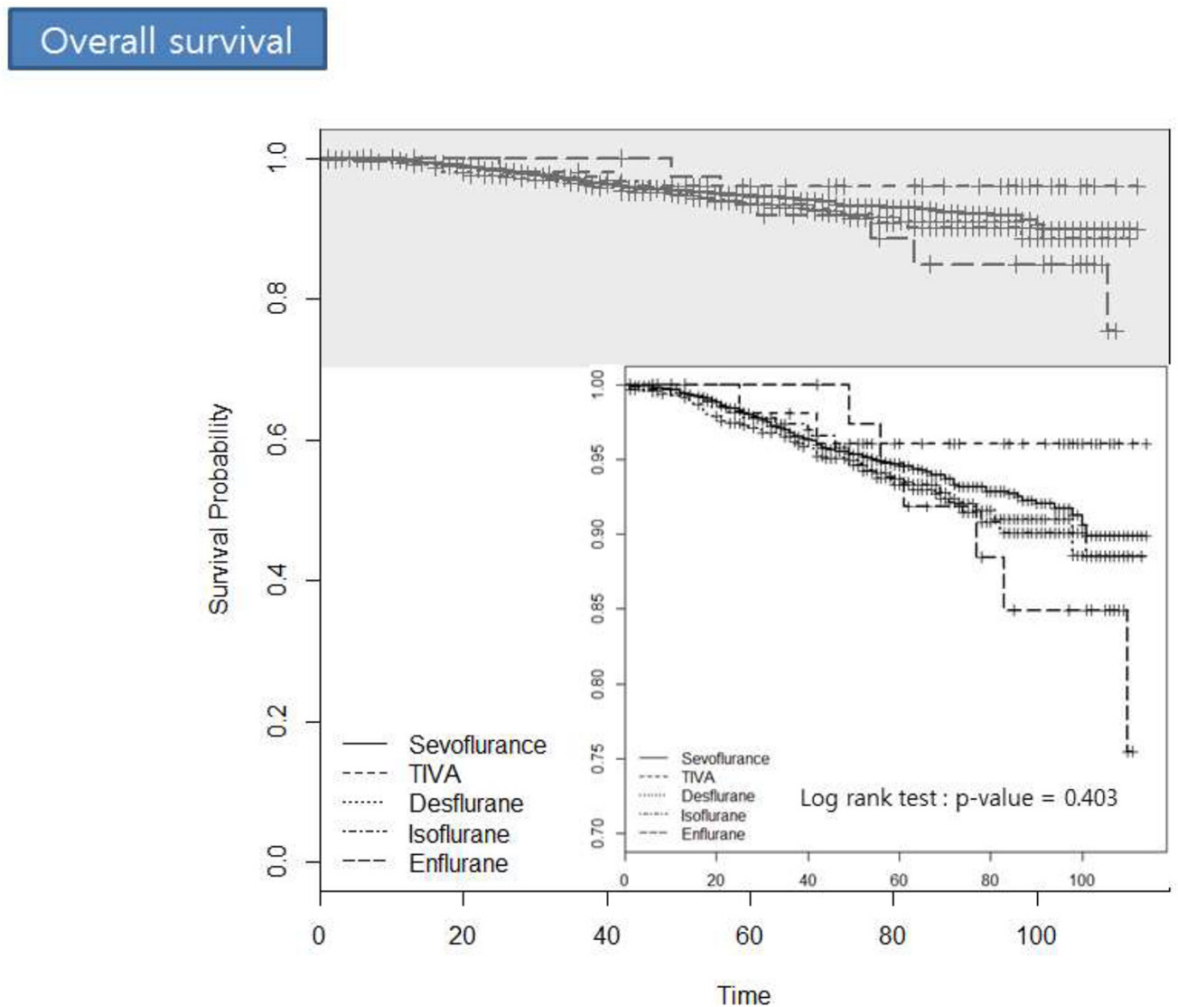
The survival probability for each anesthetic agent with regard to postoperative overall survival in breast cancer patients

Table 4 details the relationship between each anesthetic agent and overall mortality as determined by multivariate Cox regression analyses and 1:5 propensity score matching. None of the anesthetic agents demonstrated a significant association. Figure 2 demonstrates the survival probability for each anesthetic agent with regard to postoperative overall survival for breast cancer patients. Log-rank tests showed no significant differences between agents (*P* = 0.403).

**Table 4:** Association between anesthetic agents and overall mortality in patients with breast cancer after multivariate Cox regression analyses and propensity score matching

Parameter		HR	95% CI		*P*-value
Type of Anesthetic agent	TIVA	1 (ref)			
	Volatile agents	2.967	0.721	12.216	0.132
Type of Anesthetic agent	TIVA	1 (ref)			
	Sevoflurane	2.748	0.664	11.369	0.163
	Desflurane	3.277	0.777	13.834	0.106
	Isoflurane	3.457	0.795	15.030	0.098
	Enflurane	4.073	0.807	20.568	0.089
1:5 matching	TIVA	1 (ref)			
	Sevoflurane	2.848	0.357	22.713	0.323
1:5 matching	TIVA	1 (ref)			
	Desflurane	9.755	0.886	107.437	0.063
1:5 matching	TIVA	1 (ref)			
	Isoflurane	-	-	-	-
1:5 matching	TIVA	1 (ref)			
	Enflurane	-	-	-	-

HR: hazard ratio, CI: confidence interval, TIVA: total intravenous anesthesia (with propofol)

*Multivariate Cox regression analyses were adjusted by age, body mass index (BMI), antiemetic, rescue analgesics, surgery type, tumor–node–metastasis (TNM) stage, estrogen receptor status, and chemotherapy use. The statistical significance of differences in the postoperative mortality between anesthetics was tested prior to matching, and the results demonstrated that, when propofol was used as a reference, the other anesthetics showed increased overall mortality. However, the differences were not statistically significant.

**For more sensitive and precise analysis, the relative risk of overall mortality after breast cancer surgery was calculated after applying 1:5 propensity score matching after adjustment for age, body mass index, hypertension, diabetic mellitus, cardiac disease, pulmonary disease, endocrinal disease, renal disease, liver disease, neurological disease, and tumor–node–metastasis stage. The results showed no statistically significant differences between TIVA and the other anesthetics. Because the frequency of enflurane and isoflurane use was low, event occurrence after matching did not meet the minimum number requirement. Therefore, the relative risks were not calculated.

## DISCUSSION

To the best of our knowledge, this is the first study to analyses the effects of anesthetics on postoperative breast cancer recurrence and overall mortality in the long term and determine whether TIVA is effective in the prevention of recurrence compared with volatile agents, including sevoflurane, desflurane, isoflurane, and enflurane. Our findings suggest that there are no significant differences between TIVA and balanced anesthesia with each volatile agent with regard to the prevention of long-term recurrence.

The concept that the perioperative environment affects the survival after cancer surgery was suggested 40 years ago. It was reported that ether induced a greater secretion of stress hormones than halothane, reduced patient immunity, and therefore has negative effects on patient survival after surgery [11]. Although surgery is the most effective treatment for solid tumors, surgical manipulation leads to a risk of tumor spreading. Even after complete excision, the tumor cells released during surgery may eventually lead to recurrence if they bypass the immune system [12]. After complete excision, if the immune system is functioning normally, almost all the residual tumor cells are destroyed within 24 hours [13]. Unfortunately, perioperative immunosuppression inevitably starts early after surgery, and lasts for almost 7 days. This can allow tumor cells to escape from the immune system [14]. Cell-mediated immunity plays the predominant role in anti-tumor immunity, and involves natural killer cells and T lymphocytes. In the innate immune system, NK cells act as the first line of defense, while T lymphocytes activate the perioperative immune response as the secondary defence [15]. Although their effects are weaker than that of ether, isoflurane and halothane are known to decrease NK cell cytotoxicity [16]. Sevoflurane [17] and nitrous oxide [18] has also been reported to suppress tumor surveillance, including neutrophil chemotaxis. Moreover, interferon stimulates NK cell activity, an effect that is attenuated in the presence of halogenated anesthetics [16]. In addition, isoflurane has been found to increase hypoxia-inducible factor-1a and its downstream effectors to promote survival in renal cell carcinoma [19]. For this reason, the role of the anesthetist in improving long-term outcomes after cancer surgery is gaining importance. Although controversial, increasing retrospective and preclinical data have implicated inhalational anesthesia and opioid analgesia as independent risk factors for disease recurrence, whereas loco-regional anesthesia and propofol-based TIVA have been considered to have potential chemo-preventative effects [20]. Prospective randomized controlled trials on this topic are ongoing.

Propofol aids in the preservation of NK cell activity and increases cytotoxic T-cell activity, thus facilitating host resistance to recurrence and metastasis. Furthermore, it exhibits anti-inflammatory properties that could indicate greater host defense against disease recurrence in the perioperative period. These observations are supported by clinical findings [21]; therefore, the benefits of TIVA may be attributed to the facilitation of perioperative immune function. Propofol also has been found to inhibit hypoxia-inducible factors in prostate cancer [22], in accordance with the predominantly anti-cancer effects of TIVA reported in the literature. In preclinical studies, propofol inhibited cancer cell invasion [23], prevented metastasis [24], inhibited esophageal cancer proliferation and migration [25], and induced apoptosis in non-small cell lung cancer, colon cancer, and ovarian cancer cells [26]. Ke et al. [27] showed that combined propofol and remifentanil use in patients undergoing open cholecystectomy resulted in an increase in IL-10, an anti-inflammatory cytokine, as opposed to the isoflurane. A retrospective analysis of breast, colon, and rectal cancer surgeries under TIVA or sevoflurane anesthesia found improved 1- and 5-year overall survival rates of 4.7% and 5.6%, respectively [28].

Although volatile anesthetics are expected to adversely affect prognosis, including cancer recurrence, the evidence from *in vitro* studies regarding the potential deleterious effects of volatile agents is still conflicting. Moreover, there were no available data comparing the effects of TIVA and balanced anesthesia on long-term recurrence and survival. Thus, evidence to justify the avoidance of these agents in cancer patients is inadequate [19, 29, 30]. Our results suggest that there is no difference between TIVA and balanced anesthesia with each volatile agent with regard to the prevention of long-term recurrence and mortality in patients with breast cancer. However, as noted above, propofol tends to preserve immunity in comparison to sevoflurane or isoflurane in clinical trials as well as in animal studies. For this reason, we speculate that several factors account for the results of our study. First, not only propofol but also other many medications were administered during the perioperative period. Furthermore, our retrospective study design did not permit accurate quantification of intraoperative remifentanil use, although it is considered that the total remifentanil dose infused during TIVA would be greater than that infused during inhalation anesthesia, and that the efficacy of propofol may have been offset by opioid usage. There is a strong suggestion from both retrospective clinical trials and experimental studies that opioids, which suppress immune function, may promote cancer progression and decrease long-term survival [31]. Most importantly, the effects of propofol on cancer recurrence may be relatively negligible compared with those of strong outcome predictors, such as the TNM stage. The reason for the reliability why our results can obtain reliability is that, previously known as predictors [31, 32], a higher TNM staging was found to be the strong surgical risk factor for breast cancer recurrence, and ER positivity has been shown to lower risk of recurrence in our study. Further, the previous animal and short-term follow-up immunity-based clinical studies used a high concentration of volatile anesthetics alone for the induction and maintenance of anesthesia, which is no longer common clinical practice. On the other hand, the concentration of the volatile anesthetic agents analyzed in this study were set at more clinically relevant levels. Although this is a retrospective study, it is the first long-term follow-up clinical report to make such a comparison in breast cancer between TIVA and balanced anesthesia with volatile agents. Therefore, further prospective randomized clinical trials with long-term follow-up should be conducted that analyses all variables, including opioid usage, to clarify the effects of TIVA during cancer surgery on postoperative recurrence and mortality.

There are several limitations in this study. First, as this is a retrospective study, the patients were not randomized and clinical care was not standardized. Thus, selection bias and the effects of unmeasured confounding variables cannot be excluded. Nevertheless, our data were supplemented, to some extent, by the retrospective study design, because all patients were perioperatively managed in a similar manner in the same hospital for 5 years. Furthermore, the method of anesthesia was relatively consistent. Second, although a large number of patients were analyzed, the number of patients who received TIVA was small (56/2645). However, we adjusted this factor by sensitivity analysis with propensity score matching, which revealed no differences between TIVA and balanced anesthesia with volatile agents with regard to postoperative breast cancer recurrence.

In conclusion, our findings demonstrated that the type of general anesthesia, whether TIVA or balanced anesthesia with various volatile agents, has no association with the postoperative recurrence-free survival and overall survival in patients with breast cancer. However, anesthetic agents are administered at a point of potentially high vulnerability in terms of the dissemination and establishment of metastasis; therefore, there is a consistent need to determine the most appropriate anesthetic strategy for oncological surgeries in order to maximize long-term outcomes.

## MATERIALS AND METHODS

The Institutional Review Board and Hospital Research Ethics Committee of Severance Hospital, Yonsei University Health System approved this study. The requirement for informed consent was waived by the institutional review board owing to the retrospective nature of the study. The medical records of all patients who underwent breast cancer surgery at the Breast Cancer Centre between November 2005 and December 2010 were retrospectively reviewed. The follow-up period ended in December 2015.

### Anesthetic management

Anesthesia was induced by bolus administration of propofol (1–2 mg kg^−1^) or pentothal sodium (4–5 mg kg^−1^) and remifentanil (1–2 μg kg^−1^). A neuromuscular blocking agent such as rocuronium, vecuronium, or atracurium was injected to facilitate tracheal intubation. Anesthesia was maintained by balanced anesthesia with volatile agents such as sevoflurane, desflurane, isoflurane, or enflurane with adjuvant intravenous infusion of remifentanil, or performed using TIVA with 2% propofol and remifentanil. Thirty minutes before surgery completion, fentanyl (1 μg kg^−1^) was injected for postoperative pain control. Antiemetic agents were selected as per the anesthesiologist's preference.

### Clinico-pathological parameters

Clinico-pathological information, including the expression of ER, PR, and human epidermal growth factor receptor 2 (HER2), was obtained through medical records and the registry database. The registry database collected personal history, clinic-pathological parameters, treatment patterns, and follow-up outcomes associated with breast cancer. After definitive surgery for the breast and axilla, adjuvant treatments were administered according to the patients’ tolerance levels. Clinical follow-up assessments included history-taking, physical examination, laboratory tests, and imaging every 6–12 months for relapse detection. The TNM stage was determined using the American Joint Committee on Cancer 7th edition criteria. Tumors with ≥ 1% cells with nuclear staining were considered positive for ER and PR, according to the American Society of Clinical Oncology/College of American Pathologists (ASCO/CAP) guidelines [33]. Loco-regional recurrence was defined as tumor recurrence in the ipsilateral breast, chest wall, and regional lymph nodes. Any recurrence at a distant site, including the contralateral axillary or supraclavicular lymph nodes, was considered distant metastasis. Cancer recurrence was evaluated from the date of the first curative surgery to the date of the first loco-regional or distant recurrence. Overall survival was calculated from the date of the first surgery to the date of the last follow-up or death from any cause.

### Data collection

Our primary outcome was the effect of anesthetic agents on postoperative breast cancer recurrence. We collected baseline demographic data, including age, body mass index, and comorbidities, and anesthetic data, including induction agents, maintenance anesthetics, muscle relaxants, and usage of antiemetic and rescue analgesics, among others. We also documented surgical data, including the surgery type, surgical duration, TNM stage, receptor status, histological type, tumor type, and chemotherapy or radiotherapy use.

### Statistical analysis

Patients with and without breast cancer recurrence were compared for potential confounders using the x^2^ test for categorical variables and the *t*-test for continuous variables. Continuous data such as age and body mass index (BMI) were categorized by drawing a smooth hazard ratio curve because there was no linear relationship with breast cancer recurrence. To identify the association between independent variables and the primary endpoint, we used a multidimensional approach. The univariate association between recurrence-free survival (and overall survival) and anesthetic agents was assessed using the Kaplan–Meier survival estimate, and the different anesthetic groups were compared using the log-rank test. Univariate and multivariate associations between postoperative breast cancer recurrence and all potential baseline confounders were assessed using Cox proportional hazards regression. First, univariate regression analysis was performed to identify significant risk factors for recurrence. Then, variables with a *P*-value of < 0.1 were included in a multivariate regression analysis. Hazard ratios (HRs) and associated 95% confidence intervals (CIs) were estimated. Potential confounders for analysis were selected according to the literature. We also performed sensitivity analysis for our chosen method (multivariate modelling) to assess the robustness of our findings regarding the association between the anesthetic agents and recurrence (and mortality), wherein we adjusted for confounding using propensity score matching. An independent *t*-test or a x^2^ test, with a significance level of 0.05, was performed to identify variables causing statistical differences between TIVA and volatile anesthetics. The variables used for matching were age, BMI, hypertension, diabetic mellitus, cardiac disease, pulmonary disease, endocrinal disease, renal disease, liver disease, neurological disease, and tumor–node–metastasis stage, and the propensity score was calculated using logistic regression analysis. A greedy heuristic approach was used to find the optimally matching group without any loss due to statistical power or matching. The greedy heuristic approach involves a method where cases with differences exceeding twice the standard deviation (SD) during the process of matching similar propensity scores are not matched. Accordingly, 1:5 matching was found to be the most effective. The *P*-value of < 0.05 was considered statistically significant. All statistical analyses were performed with SAS version 9.4 (SAS Institute Inc., Cary, NC, USA), and Kaplan–Meier curves were analyzed using R-package version 3.0.2 (www.r-project.org).

## Abbreviations

BMI: body mass index
CI: confidence interval
ER: estrogen receptor
HER2: human epidermal growth factor receptor 2
HR: hazard ratio
NK: natural killer
PR: progesterone receptor
TIVA: total intravenous anesthesia
TNM: tumor-node-metastasis
